# Direct‐Print 3D Electrodes for Large‐Scale, High‐Density, and Customizable Neural Interfaces

**DOI:** 10.1002/advs.202408602

**Published:** 2024-11-26

**Authors:** Pingyu Wang, Eric G. Wu, Hasan Uluşan, Eric Tianjiao Zhao, A.J. Phillips, Alexandra Kling, Madeline Rose Hays, Praful Krishna Vasireddy, Sasidhar Madugula, Ramandeep Vilkhu, Andreas Hierlemann, Guosong Hong, E.J. Chichilnisky, Nicholas A. Melosh

**Affiliations:** ^1^ Department of Materials Science and Engineering Stanford University 350 Jane Stanford Way Stanford CA 94305 USA; ^2^ Department of Electrical Engineering Stanford University Stanford University 350 Jane Stanford Way Stanford CA 94305 USA; ^3^ Department of Biosystems Science and Engineering in Basel ETH Zürich Basel Switzerland; ^4^ Department of Chemical Engineering Stanford University 350 Jane Stanford Way Stanford CA 94305 USA; ^5^ Department of Neurosurgery Stanford University 350 Jane Stanford Way Stanford CA 94305 USA; ^6^ Department of Bioengineering Stanford University 350 Jane Stanford Way Stanford CA 94305 USA; ^7^ School of Medicine Stanford University Stanford University 350 Jane Stanford Way Stanford CA 94305 USA; ^8^ Hansen Experimental Physics Laboratory Stanford University 350 Jane Stanford Way Stanford CA 94305 USA

**Keywords:** 2‐photon polymerization, 3d microelectrodes, bioelectronics, retinal interfaces

## Abstract

Silicon‐based microelectronics can scalably record and modulate neural activity at high spatiotemporal resolution, but their planar form factor poses challenges in targeting 3D neural structures. A method for fabricating tissue‐penetrating 3D microelectrodes directly onto planar microelectronics using high‐resolution 3D printing via 2‐photon polymerization and scalable microfabrication technologies are presented. This approach enables customizable electrode shape, height, and positioning for precise targeting of neuron populations distributed in 3D. The effectiveness of this approach is demonstrated in tackling the critical challenge of interfacing with the retina—specifically, selectively targeting retinal ganglion cell (RGC) somas while avoiding the axon bundle layer. 6,600‐microelectrode, 35 µm pitch, tissue‐penetrating arrays are fabricated to obtain high‐fidelity, high‐resolution, and large‐scale retinal recording that reveals little axonal interference, a capability previously undemonstrated. Confocal microscopy further confirms the precise placement of the microelectrodes. This technology can be a versatile solution for interfacing silicon microelectronics with neural structures at a large scale and cellular resolution.

## Graphical abstract

This paper presents a method for fabricating 3D microelectrode arrays directly onto silicon microelectronics using two‐photon polymerization. These customizable, high‐density electrodes enable precise targeting of neurons in 3D. The technology offers scalable, large‐area neural recordings while targeting specific cells and cellular compartments, paving the way for enhanced neural prosthetics and broader applications in neuroengineering.

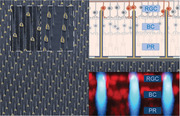

## 1 Introduction

With rapid developments in neuroelectronics and optogenetics, it is now possible to record and/or stimulate electrical activity in hundreds of neurons simultaneously.^[^ ^1^, ^2^, ^3^ ^]^ This enables investigations into the mechanisms behind neural functions such as motor control and decision‐making, as well as the development of next‐generation neural prosthetics with improved performance (e.g., speech prostheses enabled by decoding high‐density neural activity in the sensorimotor cortex).^[^ ^4^, ^5^, ^6^, ^7^ ^]^ Neuroelectronics is advantageous for developing such technologies compared to optogenetics because it does not require genetic modification, it relies on simpler setups for signal readout and write‐in, and can be applied to most parts of the nervous system.^[^ ^8^ ^]^ More importantly, thanks to advanced silicon processing, planar arrays with thousands of microelectrodes packed at a spatial density close to that of neurons have recently been demonstrated,^[^ ^9^, ^10^, ^11^ ^]^ a significant leap from previous‐generation technologies with a little over one hundred channels and coarsely spaced microelectrodes.

However, neurons are typically organized in 3D structures that are difficult to address with planar silicon microelectronic arrays. Recent innovations to bridge this geometric mismatch include fabricating complementary metal oxide semiconductor (CMOS) circuits into shanks that can be inserted into neural tissues,^[^ ^10^, ^12^ ^]^ connecting planar electronic arrays to insertable microelectrodes,^[^ ^13^, ^14^, ^15^, ^16^, ^17^, ^18^ ^]^ growing silicon or metal pillars as microelectrodes onto CMOS arrays,^[^ ^19^, ^20^, ^21^ ^]^ and patterning polymer or oxide pillars that are then metalized to become microelectrodes.^[^ ^22^, ^23^, ^24^ ^]^ These technologies leverage the spatial resolution and scalability of microfabrication processes, but unfortunately do not allow a high degree of flexibility or customizability for interfacing to diverse neural populations.

Retinal implants for vision restoration are a key example of a neural interface in which the spatial location of recording/stimulating microelectrodes needs to match the 3D organization of the target neurons. In normal vision, the retinal circuitry extracts information about the visual scene and encodes it into the spatiotemporal patterns of the retinal ganglion cells (RGC) activity known as the RGC “neural code”.^[^ ^25^, ^26^ ^]^ High‐acuity artificial vision thus requires understanding and reproducing this neural code through electrical recording and stimulation at high density. This requires precise placement of the microelectrodes into the RGC layer to avoid unwanted activation of axon bundles.^[^ ^27^, ^28^ ^]^ Additionally, the contour of the microelectrode array should conform to the natural curvature of the retina, and would ideally be customizable to the individual retina to be implanted. Finally, the electrodes should be able to target the multiple layers of RGCs present around the fovea, requiring the microelectrodes to be of different heights at different regions. The need for high‐density recording sites over a 2D area makes this a good match for using planar silicon‐based microelectronics, yet a multi‐layered microelectrode array is challenging to fabricate using traditional silicon processing schemes.

With its flexible ability to create customizable structures, additive manufacturing (also known as 3D printing) has recently been explored to fabricate neural interfacing microelectrodes.^[^ ^29^, ^30^, ^31^, ^32^ ^]^ However, these technologies require high processing temperatures and have coarse spatial resolution, limiting their ability to fabricate high‐density microelectrode arrays and applicability for silicon microelectronics. The recently developed 2‐photon polymerization (2PP) forms structures by crosslinking photosensitive polymers through simultaneous 2‐photon absorption.^[^ ^33^ ^]^ This allows 3D printing with sub‐micron resolution, a two‐order‐of‐magnitude improvement from current single‐photon technologies such as stereolithographic apparatus (SLA), making it an ideal method for directly printing high‐density, individually customizable structures as templates for microelectrodes. For example, Brown et al. recently used 2PP to fabricate high‐resolution microelectrodes onto the ends of electrical traces on flexible polymer substrates.^[^ ^34^ ^]^ These flexible devices can be implanted and recorded from the brain, but the devices are restricted in spatial density (300 µm pitch) and channel count (16) due to the need for contacting each electrode with an electrical trace on the substrate and laser micro‐machining performed on each probe to remove the passivation material.

Here, we developed a method to fabricate fully customizable and high‐density 3D arrays with 6600 microelectrodes at 35 µm pitch directly onto advanced silicon microelectronics. This was achieved by combining the customizability and high spatial resolution of 2PP, which allows precise control of the shape and height of each individual microelectrode, with scalable microfabrication processes that eliminate the need for sequential processing on individual electrodes. This process decouples the design and fabrication of the tissue‐interfacing microelectrodes from the underlying electronics, allowing for easy experimentation with microelectrode geometries and customization toward specific neural tissues. As a demonstration, we designed the microelectrode array to address the aforementioned challenge of interfacing with RGCs in the retina. We customize the height of the microelectrode array to target RGC somas while avoiding the axon bundle layer upon insertion into the retina. Successful targeting was verified with confocal microscopy and electrical recording *ex vivo*, demonstrating excellent spatiotemporal neural recordings at high density over a large area. This could be a promising solution for high‐acuity artificial vision produced by retinal prostheses—the high density and customizability of the array could allow precise activation of RGCs *en masse* to reproduce the natural RGC neural code.^[^ ^27^ ^]^ Additionally, we believe the application of this technology can be extended to other parts of the nervous system and may be a useful tool for precisely addressing neural circuits at high spatial resolution, enabling large‐scale neural interfacing at single‐cell and single‐cell‐type resolution.

## 2 Results

### 2.1 Scalable, High‐Density 3D Microelectrode Array Enabled by 2PP

**Figure 1:**
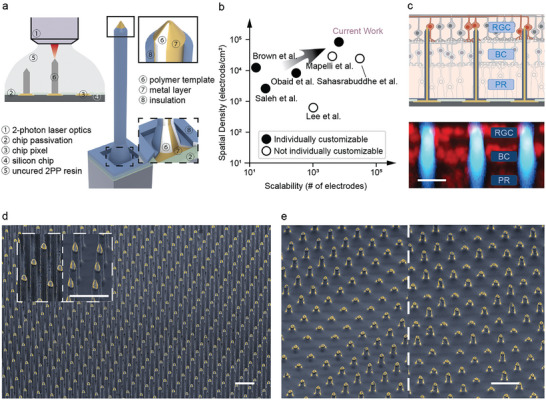
Technology overview. a) 2PP enables direct printing of 3D microelectrodes onto high‐density silicon microelectronics (④). (Left) The microelectrode templates (⑥) are first directly written. Through photolithographic patterning a conformal layer of Au (⑦), conformal insulation deposition (⑧), and selective removal of the insulation layer at the microelectrode tips, the templates are converted into individually addressable microelectrodes. (Right) 3D cutaway rendering shows the material layout of the microelectrodes. b) Our technology achieves the highest spatial density and scalability while offering customizability at single‐microelectrode level. c) With our technology, we tackle the critical challenge of precisely targeting the RGCs when interfacing with the retina. (Top) We customized the height of the microelectrodes to ensure the microelectrode tip is in proximity to RGC somas. (Bottom) Successful targeting of RGC somas is verified by confocal microscopy (red–cell nuclei, blue–microelectrodes, BC–bipolar cells, PR–photoreceptors). d) SEM images showing the highly uniform 6600‐microelectrode array that was used to record from the retina. Inset shows an example of customizing tip shapes: the conical shape could be used when easier penetration is prioritized; the hemispherical tips with less exposed area could for more localized recording. e) Our technology allows the height of each microelectrode to be varied to target neurons distributed in 3D. As an example, left shows an array with stripes of microelectrodes with varying heights (10–50 µm); right is an array of randomly distributed microelectrodes with varying heights. These arrays could be used to target RGCs around the fovea that are stacked in 3D (Images in d and e were obtained with the samples tilted at 45°; scale bars: 20 µm in (c), 50 µm in (d) and (e)).

3D printing with 2PP can fabricate customizable structures with high spatial resolution, making it an ideal method for directly printing high‐density, individually customizable microelectrodes at scale on silicon. **Figure** **1a** shows our approach to producing individually insulated microelectrodes with conductive tips and controllable height and shape. The non‐conductive core of the electrode was printed with 2PP directly onto the pixels of the silicon microelectronics array, followed by metallization, patterning, and passivation steps. The cutaway renderings show the layers of materials of the microelectrode after the fabrication process. The microelectronics array used in this work had been fabricated in CMOS 180 nm technology and features 26400 microelectrode pixels at the 17.5 µm pitch, 1024 low‐noise (<10 µV) recording channels, and 32 stimulation units.^[^ ^35^ ^]^

This combined high‐resolution 3D printing and silicon microfabrication processes achieved the highest scalability and spatial density for customizable microelectrodes compared to existing technologies (Figure 1b), as well as the capability to customize the array to target RGC somas while avoiding the axon bundle layer (Figure 1c). Figure 1d shows the fabricated high‐density (35 µm pitch, equivalating 84100 microelectrodes per cm^2^) and high‐aspect‐ratio (110 µm height, 10 µm diameter) arrays consisting of 6600 microelectrodes. We demonstrate the capability to precisely control the geometry of the microelectrodes with two examples: inset of Figure 1d shows that the shape of the electrode tips can be optimized to tailor different requirements–for example conical tips for easier tissue penetration and hemispherical tips for more localized sensing; Figure 1e shows arrays of microelectrodes with varying height, which, unlike planar arrays or protruding electrode arrays with uniform height, can be used to bypass certain structures and address neurons distributed in 3D.

### 2.2 Array Fabrication and Characterization for Neural Recording

**Figure 2:**
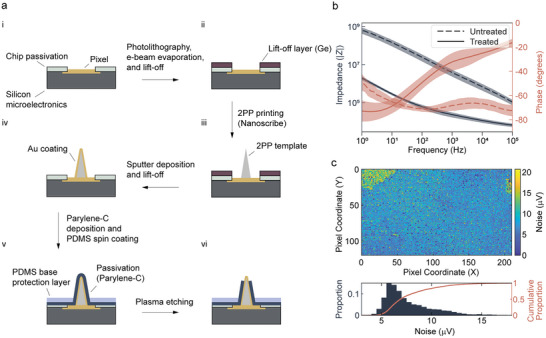
Fabrication and surface modification of the 3D microelectrode array for neural recording. a) Step‐by‐step diagram (not to scale) showing the main steps of the fabrication process. b) Bode plot (*n* = 16 microelectrodes from 2 arrays) showing the microelectrode tips can be modified post‐fabrication (e.g., electroplated with Pt‐black) to lower the electrode‐liquid interfacial impedance. Shaded region represents the standard deviation of the measured values. c) Noise map and histogram of the microelectrodes fabricated on the CMOS array. Noise distribution is highly uniform across the entire array, with anomaly at the top left and right corners caused by accidental damages to the microelectrodes during handling, as can be seen from Figure S4 (Supporting Information).

We fabricated highly uniform 3D microelectrode arrays of various designs. The fabrication process consisted of the following steps (**Figure** **2a**). The microelectrode templates were printed onto the CMOS array with 2PP, after which the device was sputter‐coated conformally with 10 nm Ti and 75 nm Au. The metal bilayer was then patterned by a Ge lift‐off sacrificial layer defined through standard photolithography prior to the 2PP step. This lift‐off process allowed each microelectrode to be electrically connected only to the CMOS pixel underneath. The microelectrode‐CMOS assembly was then passivated by Parylene‐C, and the tips were exposed at scale by oxygen plasma etching. This process was highly uniform, showing less than 5% variation in the area of the exposed tips. Despite the small size and high aspect ratio of the 2PP structures, they withstood the mechanical and thermal stress along the fabrication process, allowing highly uniform arrays to be fabricated. See Experimental Section, **Table** **1** for the detailed recipe of the fabrication process, and Figure S1 (Supporting Information) for the scanning electron microscopy (SEM) images of the sample after the main fabrication steps. We then characterized and modified the electrode‐liquid interfacial impedance of the microelectrodes to ensure they match the impedance requirement of the CMOS circuitry.^[^ ^35^ ^]^ We first measured the impedance with electrochemical impedance spectroscopy (EIS). As EIS cannot be directly performed on individual electrodes on the CMOS array due to circuitry limitations, we separately fabricated the microelectrodes on a 16‐channel planar microelectrode array (16MEA, shown in Figure S2, Supporting Information) using the same processes described above. The microelectrodes showed a high amplitude of impedance at 1 kHz (*|Z|_1k_* = 3.8 MOhm ± 1.5 MOhm) due to the small surface area of the exposed tips and the relatively high electrochemical impedance of gold. To lower the impedance, we electroplated platinum‐black (Pt‐black) onto the tips of the microelectrodes, which lowered *|Z|_1k_* by 2 orders of magnitude (*|Z|_1k_* = 23 kOhm ± 6.7 kOhm). Figure 2b is the Bode plot showing the EIS measurements of the microelectrodes before and after electroplating Pt‐black. Figures S2 and S3 (Supporting Information) show the set‐up of EIS measurement and Pt‐black electroplating, and SEM image of the electrode tip coated with Pt‐black.

**Table 1:** Fabrication Process of the Direct‐Print Microelectrodes onto CMOS Arrays.

Step		Equipment and relevant parameters
Lift‐off layer patterning	Substrate (CMOS array) preparation and priming	1) Sequential rinse with acetone, methanol, and isopropyl alcohol 2) YES hexamethyldisilazane prime oven, TA series (Yield Engineering Systems, Inc.), 150 °C, dehydration bake followed by 300‐sec vapor prime
	Spin coat photoresist (Shipley SPR 220–7)	3) Headway spin coater (Headway Research, Inc.), 3500 RPM, 50 sec 4) Soft bake at 115 °C, 5min
	Exposure	5) Heidelberg MLA 150 (Heidelberg Instruments), dose = 550 mJ/cm^2^, defocus = ‐2
	Descum	6) Samco plasma etcher (Samco, Inc.), O_2_ flow rate = 15 sccm, pressure = 15 Pa, power = 250 W, time = 3 min, plasma etch mode
	Ge evaporation	7) Innotec ES26C electron‐beam evaporator (Innotec Group Inc.), total thickness = 2 µm, evaporation rate ∼ 2.5 Å/sec
	Ge lift‐off	8) Overnight lift‐off in acetone with brief ultrasound agitation at the end 9) Rinse with acetone, methanol and isopropyl alcohol
2PP and metalization of microelectrode templates	Substrate preparation and priming	10) Samco plasma etcher (Samco, Inc.), O_2_ flow rate = 15 sccm, pressure = 15 Pa, power = 250 W, time = 3 min, plasma etch mode 11) Soaking with silanizing solution, 200:1 ethanol and 3‐(trimethoxysilyl)propyl methacrylate (440 159, Sigma‐Aldrich), 2 h
	2PP and developing	12) Nanoscribe Photonics GT (Nanoscribe GmbH & Co. KG), IP‐S kit 13) Sequential soaking in 1‐methoxy‐2‐propyl acetate and isopropyl alcohol bath for 25 min and 5 min, respectively
	Metalization	14) Samco plasma etcher (Samco, Inc.), O_2_ flow rate = 15 sccm, pressure = 15 Pa, power = 250 W, time = 3 min, plasma etch mode 15) Lesker sputtering system (Kurt J. Lesker Company), 10‐nm Ti (200 W, direct current supply, 3 mTorr) film for adhesion followed by 75‐nm Au film (200 W, direct current supply, 5 mTorr)
	Ti and Au lift‐off	16) Overnight soaking with 3% (v/v) H_2_O_2_ water solution, ultrasound agitation at the end
Insulation and selective exposure of microelectrode tips	Parylene‐C Passivation	17) SCS Parylene Labcoater® (Specialty Coating Systems). Vaporizer heater temperature = 175 °C, pyrolysis heater temperature = 690 °C, chamber pressure = 26 milliTorr (3.47 Pa), dimer amount = 4 g, resulting film thickness ∼2 µm
	Polydimethylsiloxane spin coating and curing	18) Samco plasma etcher (Samco, Inc.), O_2_ flow rate = 15 sccm, pressure = 15 Pa, power = 250 W, time = 3 min, plasma etch mode 19) Sylgard 184 (Dow), 6000 RPM, 15 min 20) Curing at 100 °C for 1 h
	Exposing microelectrode tips	21) Plasma‐ThermVersaline LL inductively coupled plasma dielectric etcher (Plasma‐Therm), inductively coupled power = 400 W, bias power = 50 W, chuck temperature = 5 °C, pressure = 3 mTorr, helium cooling pressure = 4000 mTorr

After developing this modification process, we verified that it can be used on the CMOS array fabricated with the microelectrodes, and the CMOS array remains fully functional after the fabrication and modification processes. The pixels on the CMOS array were connected to an external current source to apply the Pt‐black electroplating current to the microelectrode tips. Uniform electroplating was confirmed by noise and signal attenuation measurements in phosphate‐buffered saline (PBS, 1X). The configuration for electroplating and optical microscopy images of the electrode tips coated with Pt‐black are available Figures S2 and S3 (Supporting Information). Noise after electroplating was calculated as the root mean square (RMS) value of a blank recording band passed between 300 and 4000 Hz. Figure 2c shows the noise distribution of one array with 85.2% of the microelectrodes having noise below 10 µV. Signal attenuation was measured by injecting a 5 mV sinusoidal signal at 1 kHz into the PBS through a Pt electrode connected to a function generator and measuring the amplitude of the recorded signal, which was determined to be minimal and highly uniform signal attenuation across the entire array (Figure S3, Supporting Information). These results deem the microelectrode array suitable for neural recording.

### 2.3 Retinal Tissue Penetration with Minimal Disturbance

High‐density electrodes often pose challenges for penetration into neural tissues because of the “bed‐of‐nails” effect that distributes the insertion pressure over the entire array and causes tissue dimpling and damage.^[^ ^36^ ^]^ Here, with the small microelectrode tip diameters (<5 µm) and low volume of tissue displacement—the volume fraction of the microelectrodes is 6.4%—we were able to penetrate retina tissue with our microelectrode array as verified by confocal microscopy. A segment of isolated macaque retina ≈2 mm in diameter was first positioned onto the microelectrode array with the photoreceptor side facing the array, and a custom‐built pressing apparatus was used to apply uniform pressure to insert the microelectrodes into the retina. After visually confirming that the retina was uniformly flattened and the microelectrodes became visible, we removed the pressing apparatus and dyed the nuclei of the retinal neurons with DRAQ5. The shorter‐wavelength auto‐fluorescence from the passivation layer of the microelectrodes allowed them to be distinguished from the cell nuclei, and Z‐stacks were obtained with confocal imaging.

**Figure 3:**
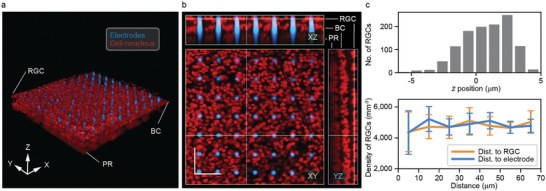
Targeted retinal tissue penetration with minimal disturbance. a) 3D rendering from a z‐stack obtained with confocal imaging. The autofluorescence of the passivation layer of the microelectrodes allow them to be distinguished from the cell nucleus that were dyed with DRAQ5^TM^. b) Orthogonal view of the *z*‐stack with the *xy* view showing the high‐density microelectrode array surrounded by RGCs. The *xz* view verifies tissue penetration and the location of the microelectrode tips being in the RGC layer. The layered structure of retinal neurons is visible in both *xz* and *yz* views. Positions of the orthogonal planes are indicated by the color‐coded lines. Scale bar: 50 µm. c) (Top) vertical distribution of RGC relative to the microelectrode tips (*z* = 0) shows that the microelectrode tips are positioned near RGC peak density. (Bottom) Density of RGCs as a function of distance to selected reference points (i.e.*, n* = 9 reference microelectrodes and *n* = 12 reference RGCs in between, error bars: standard deviation of multiple measurements across the image). There is no significant variation in RGC density as distance increases or between the two measurements with different reference points. This shows that the microelectrode array minimally disturbs RGC distribution.

The imaging results revealed that the microelectrode array through most of the retina, with the microelectrode tips precisely positioned in the RGC layer and the array causing minimal disturbance to the surrounding cells. **Figure** **3a** is a 3D rendering from a z‐stack of confocal imaging showing the retina penetration (a 3D animation is also available in the Supporting Video). The orthogonal view from the same z‐stack is shown in Figure 3b. The *xz* view confirms that the microelectrodes penetrated the retina with the tips positioned at the RGC layer. The three main layers of retinal neurons–photoreceptors, bipolar cells, and retinal ganglion cells (RGCs)–are clearly visible on both the *xz* and *yz* views. To quantify the position of the microelectrode tips relative to RGCs, we measured the positions of RGCs in the z‐stack. Figure 3c top shows the microelectrode tips were positioned near the region where RGC density peaks. Importantly, after calculating RGC density (number per mm^3^, binned every 10 µm by distance to selected microelectrode tips or RGCs in between), we found that the high density of the microelectrode array did not significantly disturb the RGC distribution as shown by the negligible variation in RGC density. This finding is consistent with the low volume displacement (6.4%) of the penetrating electrodes. It is also worth noting that the array remains largely intact after tissue penetration as can be seen in Figure 3a,b, as well as in an optical microscopy image of the array obtained after the tissue had been removed from the array (Figure S5, Supporting Information). This demonstrates the mechanical robustness of the microelectrode array.

### 2.4 High‐Density Recording from RGC Somas and Dendrites

**Figure 4:**
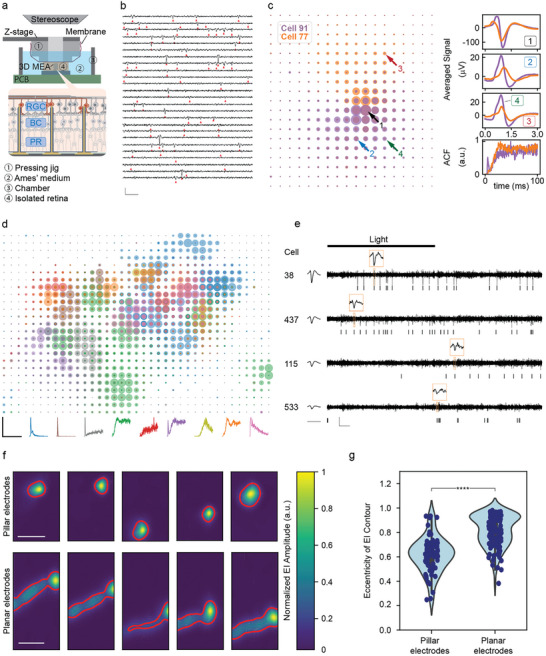
High‐density and large‐scale recording from rat RGCs *ex‐vivo*. a) Diagram of the *ex‐vivo* recording setup. A preparation of rat retina was uniformly pressed onto the microelectrode array with the setup. The microelectrode tips were positioned in the RGC layer through detection of action potentials during the pressing process. b) Waterfall plot showing exemplary band‐passed signals from 25 channels, with spiking events from corresponding channels labelled by red triangles (scale bars: horizontal– 0 ms, vertical–200 µV). c) EIs of two example cells. Each dot on the EI is centered around the microelectrode position. The sizes of the dots correspond to the amplitude of the peak voltage deflections recorded around the spiking times of a cell averaged across all spiking events. The high‐density recording allows RGC in proximity to be clearly distinguished. Cell 77 and Cell 91 have overlapping somatic activity, but different dendritic field spatial extents, as shown by their EIs and averaged voltage deflections recorded from locations indicated by the arrows. Their ACFs suggest distinct temporal activity patterns. d) Overlaid EIs of all the analyzed RGCs. The ACFs of the RGCs indicate distinct groups of temporal firing patterns, with representative ACF (normalized to respective maximum) from each group shown at the bottom. The EIs are color‐coded according to the ACFs. ACF scale bar: horizontal–120 ms, vertical–1 (unitless). e) Light stimulated neural activities of exemplary ON and OFF RGCs showing opposite changes in firing rate in response to the light stimulation. The band‐passed signal for each cell was extracted from the channel with the highest signal amplitude among all channels assigned to that cell, and was plotted in alignment with the cell's raster plot. Time‐expanded inset above each signal trace shows the waveform of a recorded action potential (scale bars for signal traces: horizontal– 00 ms, vertical–100 µV, scale bar for averaged and time‐expanded waveforms: 3 ms). f) Representative EIs obtained with pillar (top row) and planar (bottom row) arrays. The EI amplitudes were interpolated across the electrode grid and normalized, and a normalized amplitude of 0.2 was used to set the EI outlines, indicated by the red contours (scale bars: 0.5 mm) g) Violin plots showing the distributions of EI eccentricities of pillar (*n* = 67 from 2 recordings) and planar arrays (*n* = 124 from 1 recording). Lower EI eccentricities (****p < 0.0001) of the penetrating array indicates significantly reduced axonal interference.

We used the 3D array to obtain high‐density, large‐scale, and targeted recordings from the RGC layer of a 2 × 2 mm^2^ segment of an isolated rat retina. Using the same custom‐built insertion setup described above, we inserted the microelectrode array from the photoreceptor side of the retina (**Figure** **4a**). We monitored the recorded electrical activity during the insertion process. Because the photoreceptor cells and most bipolar cells of the retina do not generate action potentials, we inferred that the probe tips were near the RGCs when action potentials were observed. We then recorded spontaneous RGC activity using subsets (≈900) of the 6600‐microelectrode array at a time. Example bandpass filtered raw recordings from the electrodes are shown in Figure 4b with spiking events labeled by red triangles. The somatic spike amplitudes were in the 66.2 ± 32.0 µV range, and the typical baseline recording noise (combined instrument and physiological noise) on each electrode was roughly 19.8 ± 1.3 µV RMS. The data were spike sorted using Kilosort 2,^[^ ^37^ ^]^ and putative RGCs showing physiologically realistic refractory period and firing rate (*n* = 67) were analyzed further.

The high spatial density and scale of this recording allowed us to obtain a high‐fidelity representation of the electrical signals in each identified RGC. Using a previously reported method,^[^ ^38^, ^39^ ^]^ we calculated the electrical image (EI) of each RGC (the average spatiotemporal voltage signal recorded across the array during a spike) to identify the soma, dendrite, and axon signals recorded across the array. The autocorrelation function (ACF) of the RGC spike train was also calculated to characterize the temporal pattern of the spontaneous activity. As an example, Figure 4c shows the EIs of cell 77 and cell 91, which can be clearly distinguished despite their proximity because the somatic and dendritic signals exhibit different waveforms and amplitudes and because the two cells exhibit different dendritic field spatial extents. Additionally, the ACFs reveal different temporal patterns of activity in the two cells. The EIs of all the analyzed RGCs are plotted in Figure 4d. Notably, the RGCs in Figure 4d can be grouped according to the shapes of their ACFs, potentially revealing distinct RGC types.^[^ ^40^, ^41^ ^]^ The representative ACF in each group is shown in Figure 4d, and the EIs are color‐coded according to their corresponding ACF. We then recorded light‐stimulated RGC activities and identified ON and OFF RGCs that showed opposite responses to light stimulus. Figure 4e shows examples of ON and OFF cells’ response to light stimulation and waveforms recorded by the corresponding microelectrodes. These results demonstrate that the penetrating microelectrode array can obtain high‐quality retinal recordings with high temporal‐spatial resolution.

The EIs recorded with the penetrating microelectrode array differed significantly from those of planar electrode arrays, showing significantly reduced axonal signals. Figure 4f shows a direct comparison of example EIs from both pillar and planar arrays recorded on the same day using the same animal's retinal tissue. In EIs calculated from penetrating array recordings, we observed minimal presence of the long propagating projection representing the axonal compartment. We then quantified the presence of axonal compartments by calculating the EI eccentricity and found a significant variance between planar and pillar array recordings (Figure 4g). We attribute this to the fact that the penetrating electrode tips are closer to the RGC somas and further away from the axon bundle layer compared with planar arrays. This finding implies that cross interference with the axon bundle layer can be significantly reduced by inserting the microelectrodes specifically into the RGC soma layer. This has not been demonstrated by existing technologies and is enabled by our capability to fabricate high‐density 3D microelectrode arrays that can penetrate tens of µm into neural tissue and can be customized to address the spatial distributions of neurons in 3D.

## 3 Discussion

We demonstrated that 2PP combined with standard microfabrication processes is a versatile method for directly fabricating high‐density penetrating microelectrodes onto silicon microelectronics at scale. The method offers a high degree of customizability, which allows us to tackle the challenge of targeting RGC somas while avoiding the axon layer. We verified successful retina penetration and avoidance of the axon layer with confocal microscopy and electrical recording. The high‐quality recording with our array allows precise mapping of retinal neurons, with a spatial resolution revealing activities from various cellular compartments within a single cell.

Tissue penetration proved beneficial for RGC recordings. We demonstrated the capability to precisely position the microelectrodes in the RGC layer, thus significantly reducing axonal interference. This is presumably because penetrating microelectrode tips are closer to the neurons than planar electrodes positioned on the surface of neural tissues.^[^ ^42^ ^]^ For future retinal implant efforts aimed at vision restoration, these penetrating electrodes could be used to selectively electrically stimulate the axonal initial segment near an RGC soma and avoid inadvertently stimulating passing axons from untargeted RGCs. Combined with previous work that allows identification of RGC types based on their spontaneous activity,^[^ ^41^, ^43^ ^]^ it may then be possible to electrically stimulate with cell‐type specificity, allowing accurate reproduction of the natural RGC neural code.^[^ ^44^ ^]^

The ability to penetrate through several layers of neurons and neuropil with high‐density electrodes may prove important for interfacing with the fovea–a region of the retina consisting of RGCs densely packed in several layers and responsible for central high‐acuity vision. The flexible fabrication method can be used to address the distinct layers with electrodes of varying length, while also accommodating the complex curved surface of the foveal region. This could enable the restoration of central vision, which is critical for most visual tasks.

To the best of our knowledge, tissue penetration with 3D electrode arrays of this density has not been reported previously. We successfully inserted these high‐density microelectrodes into retinal tissue while causing minimal displacement of RGCs, resulting in high‐quality recordings. The shape of the microelectrode tips could be optimized for insertion in other applications.^[^ ^34^ ^]^ This is a direct benefit from the versatility of 2PP which is impossible to reach with conventional microfabrication processes.

2PP has shown limited application in neuroelectronics because most of the materials available for 2PP are non‐conductive polymers. Although 2PP of conducting polymers is possible, the trade‐off between conductivity and structural deformation during printing limits their potential for fabricating microelectrodes with high precision.^[^ ^45^ ^]^ Another limitation of current 2PP technologies is the requirement of direct laser writing, which can limit the scalability of the process. However, 3D microprinting based on photoresin's optical nonlinearity is a rapidly evolving technology showing great promise for improving printing speed. For example, a recent study by Hahn et al. demonstrated a light‐sheet 3D microprinting technology with significantly enhanced printing speed and micrometer‐level resolution.^[^ ^46^ ^]^ Our technology is inherently translatable to these new direct‐print technologies with higher printing speed and scalability.

Our process proves to be compatible with silicon microelectronics that are often sensitive to high temperatures and electric discharging events. The electrochemical properties of the microelectrodes were verified to be highly uniform and suitable for large‐scale neural recording. Although electroplating of platinum black was required to lower the electrode‐saline interfacial impedance, it is possible to directly deposit low‐impedance materials, such as titanium nitride or iridium oxide, during the sputter‐coating and lift‐off steps of the process. This feature makes our process completely compatible with any integrated circuits including those that do not support on‐chip electroplating.

Together these findings demonstrate that 2PP combined with standard microfabrication technologies provides a versatile method to interface advanced silicon microelectronics to neural structures. In the retina, the penetrating microelectrodes provide the capability to record (and potentially stimulate) more selectively than was previously possible.

In the future, this technology could be expanded to access other multi‐layer neural ensembles such as the brain cortex. Additionally, we believe multimodal neural interfacing is possible with our technology. For example, 2PP can be used to fabricate microfluidic channels together with the micropillars. This would enable high‐density and 3D neural interfacing using both electrical and chemical signals, which could be an emerging technology for various applications including drug screening and studying neural or disease development.

## 4 Experimental Section

### Fabrication, Packaging, and SEM Imaging of the Microelectrode Array

A total of 4 CMOS arrays were fabricated with the 3D microelectrodes. The CMOS array contains 26400 pixels at 17.5 µm pitch. The Ge lift‐off layer was defined such that four pixels were used to address one microelectrode, allowing 6600 microelectrodes to be fabricated per array. 2PP directly on the pixel was challenging due to the high reflectance of the metal surface, so each microelectrode template was printed on the passivation next to the pixel. The metalization step established an electrical connection between the microelectrode and the pixel. Table 1 summarizes the process and relevant parameters for fabricating the microelectrode array.

The CMOS arrays fabricated with the microelectrodes were wire‐bonded to custom‐printed circuit boards (PCBs) and packaged as described previously.^[^ ^35^ ^]^ Briefly, after wire bonding, a ceramic chamber ring was first positioned around the wire bonds and glued to the PCB using a two‐part epoxy (EPO‐TEK 353ND‐T, Epoxy Technology, Inc.). Then, the same epoxy was applied around the microelectrode array in the form of a 1‐mm line dispensed through a syringe. The epoxy was partially cured at 80 °C for 1 h. After the assembly cooled down from the baking, a different epoxy with a thinner consistency (EPO‐TEK 353ND, Epoxy Technology, Inc.) was poured into the space between the previously drawn epoxy line and the ceramic ring to cover the exposed wire ‐bonds, with the line drawn around the microelectrode array preventing the uncured epoxy from flowing onto the array. Finally, the epoxy was cured with a temperature‐profiled baking procedure, where the temperature was first held at 80 °C for 30 min, and then ramped to 150 °C within 30 min and held for 1 h. The assembly was slowly cooled over a few hours and ready for testing. 2 CMOS arrays were wire‐bonded and packaged for characterization and tests.

SEM imaging of the fabricated arrays was conducted with a field‐emission scanning electron microscope (Quanta 250 FEG, FEI company) using acceleration voltage = 2 kV, spot size = 3, and working distance ≈10 mm. The images were false‐colored using gradient mapping in Adobe Photoshop 2023.

### Characterization and Modification of the Microelectrode Array for Neural Recording

The CMOS array used in this work does not allow direct ohmic contact to the fabricated microelectrodes to perform EIS, so the microelectrodes were separately fabricated on the custom‐designed 16MEA using the same process described above. The microelectrodes were electrically connected to the contact pads on the periphery of the 16MEA. A total of 5 arrays were fabricated for characterization and tests.

EIS was measured with a two‐electrode configuration using a potentiostat (SP‐200, BioLogic). A droplet of PBS (1X) was positioned to submerge the microelectrode array, and a Pt electrode (327492, Sigma–Aldrich) was submerged in the same droplet and connected to the counter and reference electrode port on the potentiostat. The contact pad on the 16MEA was connected to the working electrode port to inject sinusoidal signals (amplitude = 10 mV, injected at 10 evenly spaced frequencies across each decade, total frequency range 1 Hz–100 kHz). The full‐spectrum EIS across 16 microelectrodes on the 16MEA was measured and computed using EC‐Lab (BioLogic). One microelectrode was damaged during Pt‐black electroplating and was not measured post‐electroplating.

To lower the electrode‐electrolyte interfacial impedance, Pt‐black electroplating on the 16MEA was conducted with the same configuration. Instead of PBS (1X), a droplet of Pt‐black electroplating solution containing 3 wt.% chloroplatinic acid hexahydrate (206083, Sigma–Aldrich) and 0.3 wt% lead(II) acetate trihydrate (215902, Sigma–Aldrich) was used. Electroplating was performed with a constant voltage (−1.1 V against the Pt wire counter electrode) applied to one microelectrode each time (duration was controlled by the total charge transferred *Q_TOT_* < 800 nC), and the 16 microelectrodes were electroplated sequentially. After electroplating, the array was rinsed with deionized (DI) water.

Pt‐black electroplating on the packaged CMOS array followed the same procedure as previously reported.^[^ ^35^ ^]^ In short, after filling the chamber with the Pt‐black electroplating solution, a Pt wire connected to an external current source (2410, Keithley) was submerged in the solution. Utilizing the stimulation circuity on the CMOS array, all pixels were connected to a common current sink in order to flow the electroplating current. A constant current of −0.6 mA was applied through the Pt wire into the solution for 1 min. Finally, the solution was removed and the chamber was rinsed with DI water.

Noise and signal attenuation of the microelectrodes were measured using the same method described previously.^[^ ^18^ ^]^ For noise measurement, with the chamber filled with 1X PBS, a blank recording was obtained with the amplifier gain of the CMOS array set to 512, and the noise was calculated by the root mean square amplitude of the blank recording. Attenuation measurement was conducted by injecting a 5 mV, 1‐kHz sinusoidal signal into the 1X PBS and recording with the amplifier gain set to 28. Attenuation was quantified using the relative amplitude of the recorded versus the injected signal.

### Tissue Preparation, Penetration test, and Confocal Microscopy

Totally 9 tissue penetration and imaging tests were successfully conducted. Results reported in this paper involved isolated rhesus macaque monkey (*Macaca mulatta*) retinas. Tissue preparation followed previously reported protocols^[^ ^38^ ^]^ and was in accordance with institutional and national guidelines (Stanford Administrative Panel on Laboratory Animal Care, protocol number 34387). Briefly, eyes were removed from terminally anesthetized macaque monkeys, immediately followed by the removal of the anterior portion of the eyes and vitreous. Tissues with size of 2 by 2 mm were then dissected. Perfusion with Ames’ solution (A1420, Sigma–Aldrich, 31–36 °C, pH = 7.4, bubbled with 95% O_2_ and 5% CO_2_) continued throughout the preparation.

To test and verify retinal tissue penetration, identical dummy arrays were fabricated on bare silicon substrates using steps 10–20 described above without the metal lift‐off step (step 14). The dummy array was secured to a petri dish that was used as a chamber for tissue penetration testing. The prepared retinal tissue was transferred to the testing chamber filled with Ames’ solution, and positioned onto the microelectrode array with the photoreceptor side facing downward. A custom‐built pressing apparatus consisting of a micromanipulator, a support arm, and a cylinder with nylon mesh (9313T48, McMaster‐Carr) stretched taut on the end facing the retinal tissue was used to uniformly apply pressure on the retinal tissue. While monitoring through a stereoscope, the cylinder was slowly lowered by the micromanipulator to insert the microelectrodes into the tissue from the photoreceptor side. After visually confirming that the tissue was in contact with the substrate of the dummy array, the pressing setup was carefully removed.

For confocal microscopy, the tissue embedded in the microelectrode array was first fixed with 1X PBS containing 4% paraformaldehyde (8.18715, Sigma–Aldrich), then rinsed with 1X PBS twice, each lasting 20 min, and finally stored in 1X PBS. Then, DRAQ5 (5 mM, 62251, ThermoFisher) was added to the preparation and gentle agitation was applied for 30 min. Finally, the dummy array together with the retinal tissue was added with a mounting medium (ab104135, Abcam) and prepared into a microscope slide. Confocal microscopy was performed on ZEISS Airyscan2 LSM 980 installed with a Plan‐APO 10x objective using a 639‐nm excitation laser. The signals from the retinal tissue and the microelectrodes were filtered by the program (ZEN 3.3, Blue Edition) built‐in DRAQ5 and DAPI filters, respectively. Z‐stacks with 1 µm spacing between adjacent slices were obtained from regions of interest.

Each slice of the *z*‐stack was adjusted by the “enhance contrast” process in ImageJ, with the fraction of saturated pixels set to 0.35%. The 3D rendering was generated with the “3D viewer” plugin in ImageJ. The *xz* and *yz* images of the orthogonal view were denoised with an ImageJ plugin developed by Mannam et al.^[^ ^47^ ^]^

### Ex‐Vivo Recording from Rat Retina and Calculation of Electrical Images

Rat (Long Evans, male) retinal tissue was prepared in accordance with institutional and national guidelines (Stanford Administrative Panel on Laboratory Animal Care, protocol number 28860), and the CMOS array with microelectrodes was inserted using the same method described above. To position the microelectrode tips near the RGCs, the microelectrodes were slowly inserted from the photoreceptor side while recording, and insertion stopped when putative action potentials obtained with signal thresholding were observed. Subsets (≈900) of the 6600 microelectrodes were used to record spontaneous RGC activity at a time as the CMOS array allows simultaneous recording with up to 1024 channels. The CMOS array amplifier gain was set to 1024.

Recording data analysis and electrical images of the RGCs used the method reported by Li et al. and Litke et al.^[^ ^38^, ^39^ ^]^ Briefly, the recorded signals were bandpass‐filtered with a finite impulse response filter with a passband between 300 and 6000 Hz and then spike‐sorted with Kilosort 2 to identify candidate neurons and their spiking time. The autocorrelation function and the average firing rate of the neurons were calculated, and only neurons showing refractory periods and realistic average firing rates were included in analyzing the electrical images. To compute the electrical images of an identified RGC, we averaged–across all the spike times–the signals recorded by each microelectrode within the time window (−1 to 2 ms) around the peak negative potential. The peak amplitude of the average signal is represented by the size of the discs plotted at the coordinate of each microelectrode. This generates a visual mapping showing the RGC's activity in the microelectrode space, and we repeated the calculation for all identified neurons. To outline the contours of EIs, the EI data was interpolated across the electrode grid using a linear radial basis function, as implemented in the “Rbf” function from SciPy's interpolation module. To mitigate the impact of high‐frequency noise on visualizing the EIs, the interpolated data was then smoothened using a Gaussian filter (*σ* = 2) and normalized. The Shapiro‐Wilk test and the Mann‐Whitney U test were used to assess whether the eccentricity distributions of the EI contours between the planar and pillar arrays were statistically different.

Light stimulation of the retinal tissue was performed with a white‐light flashlight, and the stimulation pulses were ≈6 s. ON and OFF RGCs were determined by their change in firing rates to the light stimulation: ON and OFF RGCs respectively increased and decreased firing rates in the presence of light stimulation. The group average firing rates were then calculated for ON and OFF RGCs with a bin size of 500 ms.

## Conflict of Interest

The authors declare no conflict of interest.

## Author contributions

P.W., N.A.M., and E.J.C. conceived the overall study. P.W., N.A.M., and G.H. designed, and P.W. performed experiments on fabricating the microelectrode array with additional advice from E.T.Z., H.U., and A.H. P.W., E.G.W., and E.T.Z. characterized the microelectrode array with advice from H.U. E.J.C., E.G.,W., A.J.P., M.R.H., P.W., A.K., S.M., R.V., and P.K.V. designed, and, E.G.,W., A.J.P., M.R.H., P.W., A.K., E.T.Z., P.K.V., S.M., and R.V. performed experiments and/or analyzed data on retina penetration, confocal microscopy, and recording. P.W., N.A.M., E.J.C., and E.G.W. wrote the manuscript with input from all other co‐authors.

## Supporting information



Supporting Information

Supplemental Video 1

## Data Availability

The data that support the findings of this study are available from the corresponding author upon reasonable request.
