# Supplementary material for: Direct‐Print 3D Electrodes for Large‐Scale, High‐Density, and Customizable Neural Interfaces
Source: Adv Sci (Weinh). 2024 Nov 26;12(3):2408602. doi: 10.1002/advs.202408602 (PMC11744676; doi:10.1002/advs.202408602)
Supplement: Supplementary file 1 — Supporting Information [file ADVS-12-2408602-s001.docx]

Supplementary Materials for

**Direct-print three-dimensional electrodes for large-scale, high-density, and customizable neural interfaces**

Pingyu Wang^[[1]](#footnote-1)^, Eric G. Wu^[[2]](#footnote-2)^, Hasan Ulusan^[[3]](#footnote-3)^, Eric Tianjiao Zhao^[[4]](#footnote-4)^, A.J. Phillips^2^, Alexandra Kling^[[5]](#footnote-5)^, Madeline Rose Hays^[[6]](#footnote-6)^, Praful Krishna Vasireddy^2^, Sasidhar Madugula^[[7]](#footnote-7)^, Ramandeep Vilkhu^2^, , Andreas Hierlemann^3^, Guosong Hong^1^, E.J. Chichilnisky^5,^ ^[[8]](#footnote-8)^, Nicholas A. Melosh^1,^ ^[[9]](#footnote-9)^*

Table of Contents

[Fabrication Process 1](#_Toc135221586)

[Electrode development for neural recording 2](#_Toc135221587)

[Packaged device and retina recording setup 4](#_Toc135221588)

# Fabrication Process


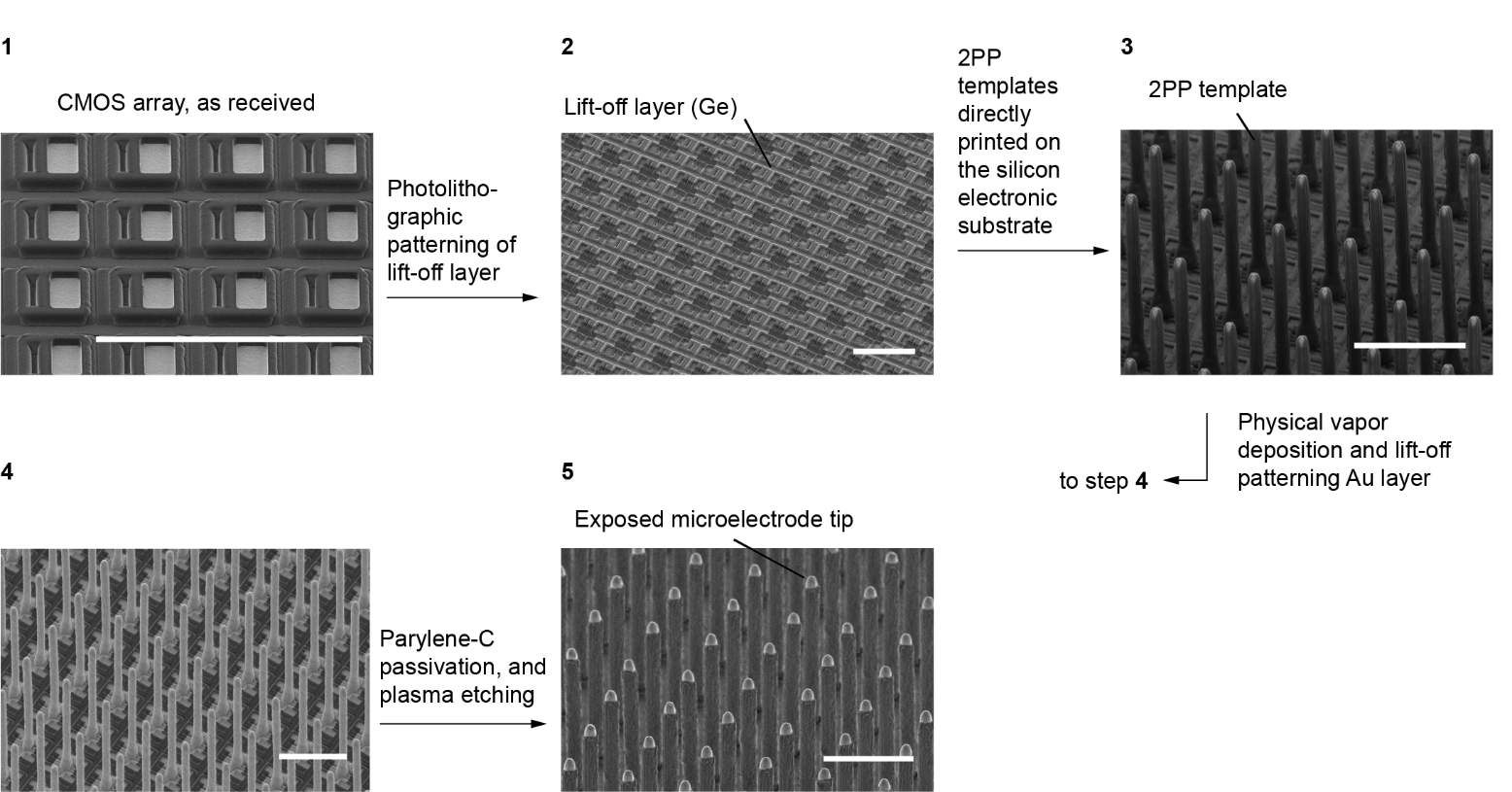


**Fig. S1** | **Sample SEM images after key fabrication steps** (scale bars: 50 μm)

# Electrode development for neural recording


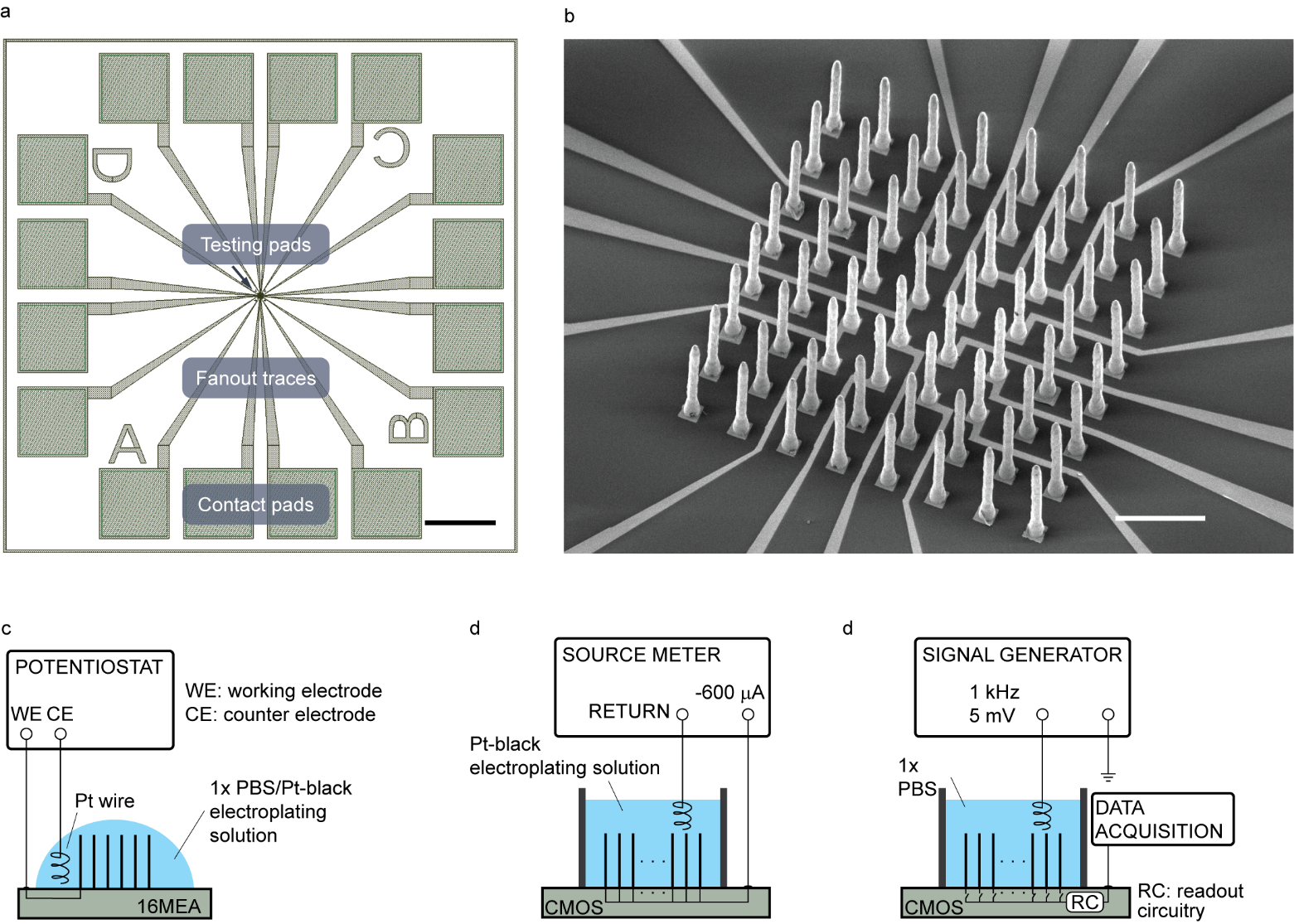


**Fig. S2** | **16MEA and setups for electrode development**

**a** Layout of testing pads and contact pads on the 16MEA. Scale bar: 3 mm. **b** SEM image showing an 8 x 8 microelectrode array printed onto the 16MEA, of which 16 are electrically connected by the fanout traces to the contact pads for testing; image was acquired after the Au lift-off step. Scale bar: 50 μm. **c** Setup for EIS and Pt-black electroplating using the 16MEA. **d** Setup for Pt-black electroplating on the packaged CMOS array printed with the microelectrode array. All microelectrodes are electrically shorted to an external source meter to allow for simultaneous electrodeposition across the entire microelectrode array. **e** Setup for signal attenuation measurement using the packaged CMOS array printed with the microelectrode array. Given the limited number of recording channels (1024), the on-chip switching circuits were used to sample tiling subsets of the 6,600 microelectrodes in order to map the attenuation of the entire array. For details of the readout circuitry, please refer to Müller *et al*., 2015 referenced in the main text.


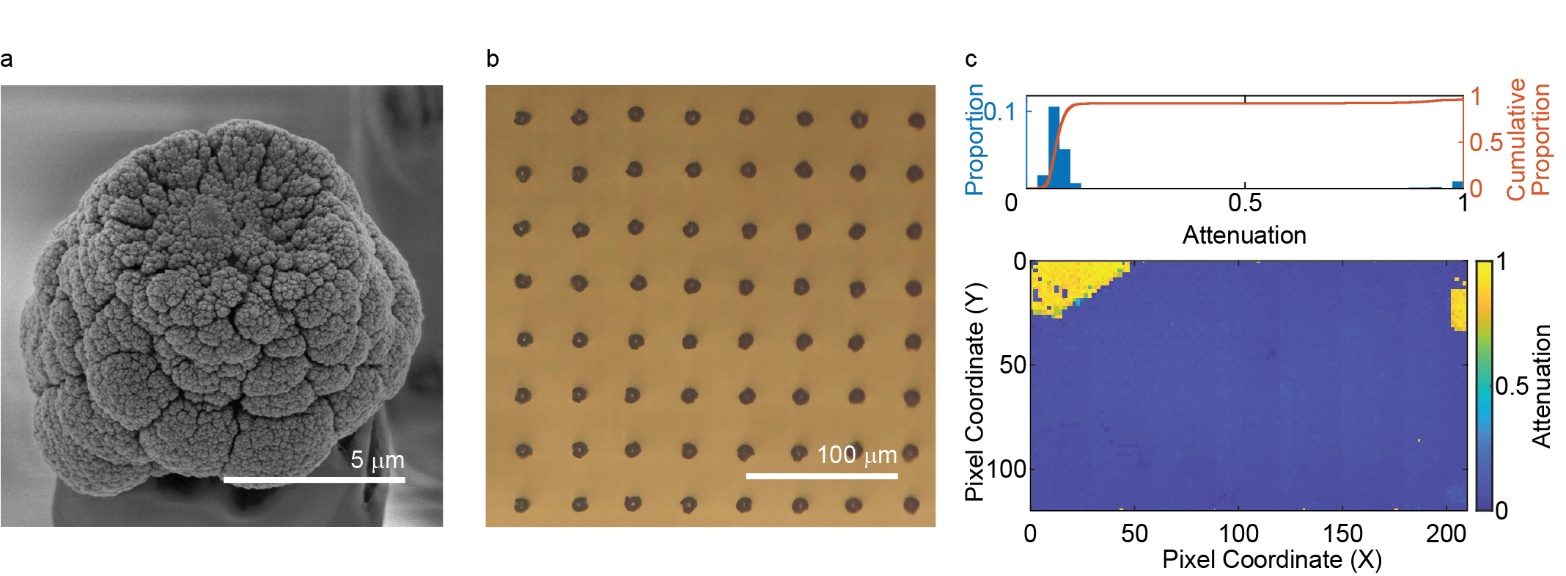


**Fig. S3** | **Electrode characterization for neural recording**

**a** SEM image and **b** optical micrograph of the microelectrode tips coated with Pt-black. **c** Signal attenuation histogram and map of the microelectrodes fabricated on the CMOS array.

# Packaged device and retina recording setup


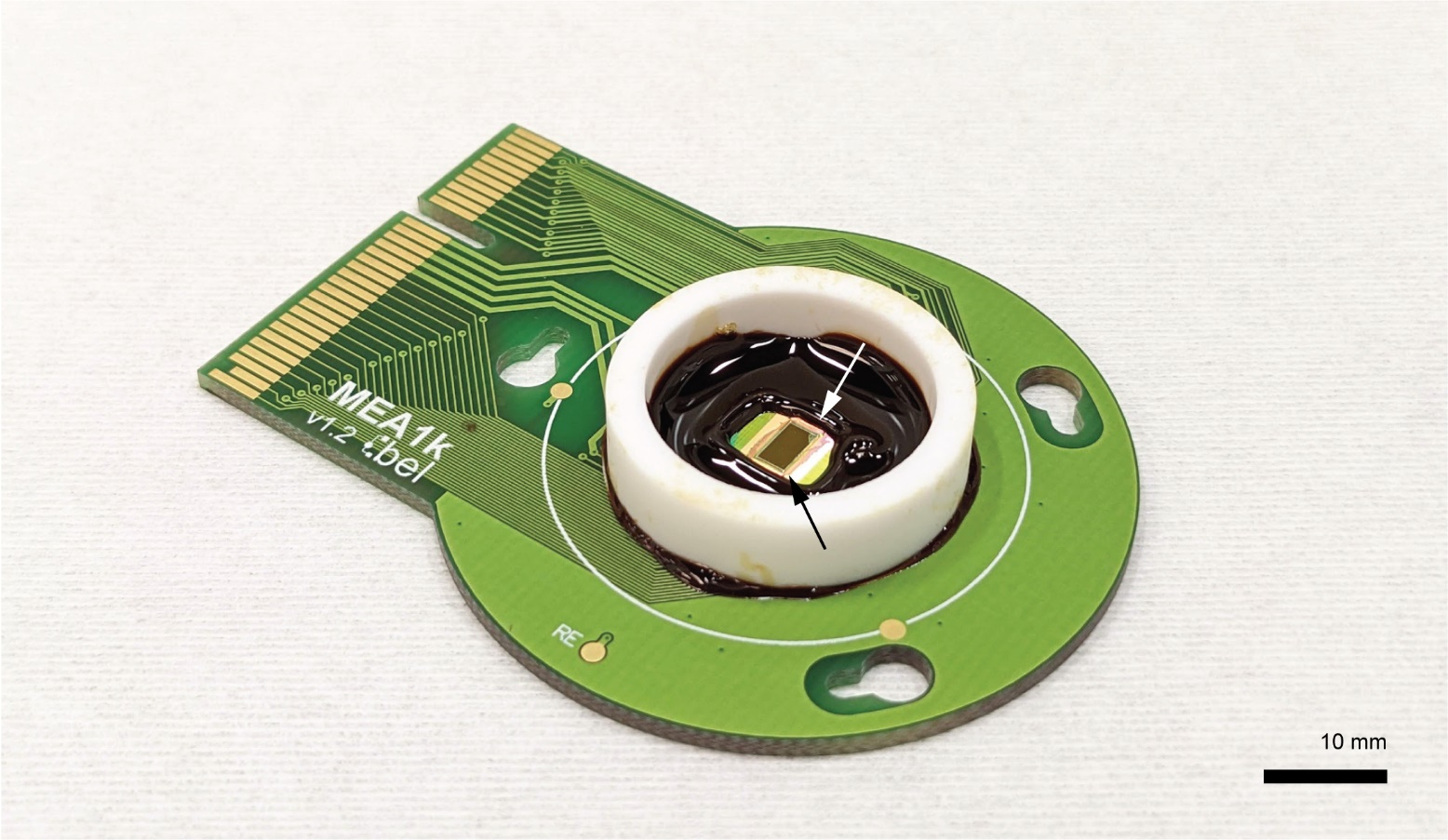


**Fig. S4** | **Packaged CMOS array**. The CMOS array printed with the microelectrodes were wire-bonded to a custom printed circuit board and the wire bonds were covered with epoxy. Arrows indicate locations where the microelectrodes were accidentally damaged during handling.


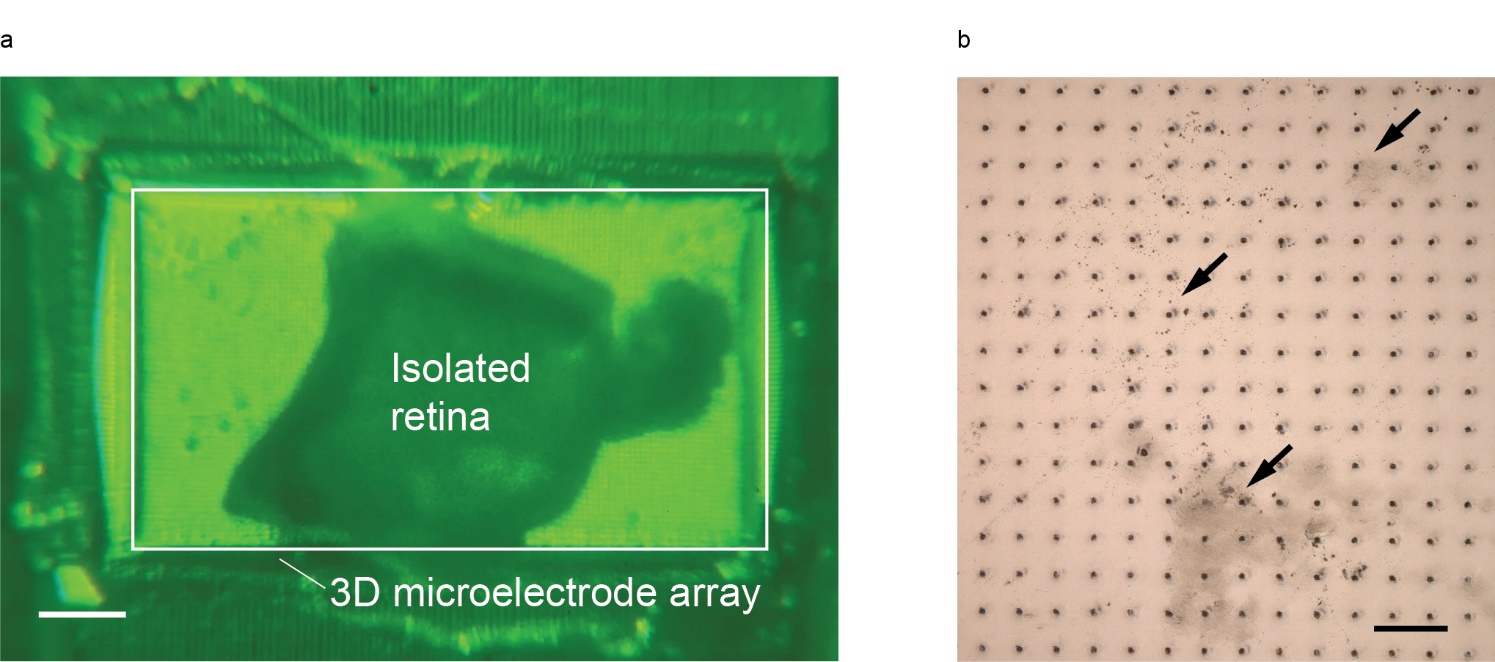


**Fig. S5** | **Retina pressing setup** **a** An image obtained with the stereoscope showing the isolated retina positioned on top of the 3D microelectrode array (outlined by the white rectangle) right before pressing. Scale bar: 500 μm. **b** An optical micrograph showing the microelectrode array remains largely intact after insertion into and removal from the retina. Residual retinal tissue (indicated by arrows) can be seen across the array. Scale bars: 50 μm.

1. Department of Materials Science and Engineering, Stanford University [↑](#footnote-ref-1)
2. Department of Electrical Engineering, Stanford University, Stanford University [↑](#footnote-ref-2)
3. Department of Biosystems Science and Engineering in Basel, ETH Zürich [↑](#footnote-ref-3)
4. Department of Chemical Engineering, Stanford University [↑](#footnote-ref-4)
5. Department of Neurosurgery, Stanford University [↑](#footnote-ref-5)
6. Department of Bioengineering, Stanford University [↑](#footnote-ref-6)
7. School of Medicine, Stanford University, Stanford University [↑](#footnote-ref-7)
8. Hansen Experimental Physics Laboratory, Stanford University [↑](#footnote-ref-8)
9. * Corresponding author, email: nmelosh@stanford.edu [↑](#footnote-ref-9)
